# The Physiological Effect of Muscular Exercise

**Published:** 1870-12

**Authors:** 


The Physiological Effect of Muscular Exercise.—In some remarks on this subject recently made before a medical society in New York, Dr. Austin Flint, Jr., stated that there is an increased quantity of excrementitious matter produced in the system on violent muscular exertion.

Some recent experiments seem to throw doubt on this statement, but he thought their results were not correct.

Within physiological limits, the elimination of this excess of effete matter is attended, if proper nutriment be supplied, by increased nutrition. If a man in health, eating and drinking according to a correct appetite, exercise his muscular system so as to increase to its highest point the elimination of effete matter, he will correspondinly increase the nutrition of his muscular system. If we can thus develop the muscular system the rest of the body will share in the improvement.

Can a man by such exercise successfully combat the tendency to deposits in his tissues, to the formation of tubercles?

Dr. F. thought such was possible; he had seen consumptives, with tubercles already formed, go to the gymnasium and become hale and robust. He knew one case in particular where the patient became an accomplished gymnast after tubercles had been clearly diagnosed in his lungs. It would seem that these men might by increasing the deposit of normal material, have removed the disposition to the deposit of that which is abnormal.

Let a man moderately healthy, work as hard as he pleases with his brain—if only he take the appropriate exercise to keep the system throwing off the effete matter, and appropriating in its place, healthy nitrogenized functioning matter, he will be comparatively safe.

It is better by constant exercise to keep ourselves healthy, than to break down every few months and have to take a vacation to recruit again.—*N. Y. Medical Journal.*

